# Biliverdin Protects against Liver Ischemia Reperfusion Injury in Swine

**DOI:** 10.1371/journal.pone.0069972

**Published:** 2013-07-29

**Authors:** Barbara Andria, Adele Bracco, Chiara Attanasio, Sigismondo Castaldo, Maria Grazia Cerrito, Santolo Cozzolino, Daniele Di Napoli, Roberto Giovannoni, Antonio Mancini, Antonino Musumeci, Ernesto Mezza, Mario Nasti, Vincenzo Scuderi, Stefania Staibano, Marialuisa Lavitrano, Leo E. Otterbein, Fulvio Calise

**Affiliations:** 1 Centre of Biotechnologies, “Antonio Cardarelli” Hospital, Naples, Italy; 2 Liver Transplantation Unit, “Antonio Cardarelli” Hospital, Naples, Italy; 3 Department of Advanced Biomedical Sciences,“Federico II” University, Naples, Italy; 4 Department of Cardiac Surgery, “Federico II” University, Naples, Italy; 5 Department of Surgery, University of Milano-Bicocca, Monza, Milan, Italy; 6 Department of Surgery, Harvard Medical School, Beth Israel Deaconess Medical Center, Boston, Massachusetts, United States of America; University of Leicester, United Kingdom

## Abstract

Ischemia reperfusion injury (IRI) in organ transplantation remains a serious and unsolved problem. Organs that undergo significant damage during IRI, function less well immediately after reperfusion and tend to have more problems at later times when rejection can occur. Biliverdin has emerged as an agent that potently suppress IRI in rodent models. Since the use of biliverdin is being developed as a potential therapeutic modality for humans, we tested the efficacy for its effects on IRI of the liver in swine, an accepted and relevant pre-clinical animal model. Administration of biliverdin resulted in rapid appearance of bilirubin in the serum and significantly suppressed IRI-induced liver dysfunction as measured by multiple parameters including urea and ammonia clearance, neutrophil infiltration and tissue histopathology including hepatocyte cell death. Taken together, our findings, in a large animal model, provide strong support for the continued evaluation of biliverdin as a potential therapeutic in the clinical setting of transplantation of the liver and perhaps other organs.

## Introduction

The increasing need of organs for orthotopic liver transplantation (OLT) has led to consider the use of marginal livers. A liver is considered marginal when obtained from a donor with hemodynamic instability prior to donation and/or aged more than 65 years. Typically the organ also exhibits a high degree of steatosis (greater than 40% macro-steatosis) and particularly, undergoes a cold ischemia time of more than 12–14 hours before reperfusion. We thus set up a preclinical model of ischemia-reperfusion injury (IRI) using organs with prolonged cold ischemia time (19 hours) to provide potentially useful information for a prompt application to clinical practice [1], [2] where there remains a desperate shortage of available organs.

Ischemia reperfusion injury in organ transplantation remains a crucial problem, especially given its association with more frequent problems later in the life following transplant [3]. Organs that undergo significant damage during IRI function less well immediately after reperfusion (delayed graft function); precipitating longer hospital stays, and have more problems in the later phases of rejection [4]. While studied most extensively with respect to organ transplantation, IRI also plagues clinical practices such as heart bypass and vascular surgery, stroke and sepsis. In all these situations there is some degree of ischemia or a hypoxic event followed by reperfusion and reoxygenation during which the majority of the damage occurs.

The pathophysiology of IRI is complex. Prominent features include oxidative stress, inflammation with infiltration of neutrophils and monocytes, cell death and ultimately loss of cell and organ function, contributing in the extreme to multi-organ failure [5], [6]. Likely because of the complexity and diversity of pathological processes that comprise IRI, no established effective pharmacological treatment has been discovered.

Heme oxygenase-1 (HO-1) and its products are accepted molecules by which to effectively treat IRI based on studies in rodents and large animals [7]. Not only does HO-1 expression lead to removal of heme, a powerful oxidant when present in excess, but the degradation of heme by HO-1 leads to the production of carbon monoxide (CO) and biliverdin that have potent anti-oxidant and anti-inflammatory effects leading to overall cytoprotection and restoration of homeostasis [8]. Degradation of heme also leads to the release of ferrous iron that stimulates the up-regulation of ferritin, an iron and heme-binding molecule that imparts protection in a rodent model of liver IRI [9]. Administration of exogenous CO or biliverdin in most cases leads to the same overall therapeutic effects as increased expression of HO-1 [10]. One or both of these molecules have been demonstrated to protect against a wide range of disorders in mice and rats including hepatitis, neointima formation after balloon injury, atherosclerosis, pulmonary hypertension, inflammatory bowel disease and several others [7], [11], [12]–[14]. With regard to transplantation in rodents, HO-1 overexpression or CO administration suppresses IRI and chronic rejection. Biliverdin administration protects in IRI but also suppresses T cell mediated acute rejection.

Considering therefore that biliverdin could offer potential therapeutic benefit in humans, we felt it important to assess these substances in an accepted pre-clinical species such as the pig. We have shown in earlier work that CO protects against IRI in pig models of cardiopulmonary bypass, paralytic ileus, delayed graft function of a kidney transplant and balloon angioplasty-induced stenosis [12]–[15]. There are no studies in pigs or any other large animal species with biliverdin. To evaluate the efficacy of biliverdin against IRI in the present study, we used a model of isolated perfused liver.

## Materials and Methods

### Animals

All studies have been approved by the IACUC at Cardarelli Hospital, Center for Biotechnology. Female Large-White pigs (20–30 kg) were purchased from a local farm. Animal care and experimental procedures met local, national, and European Union Guidelines for the use of animals.

**Figure 1:**
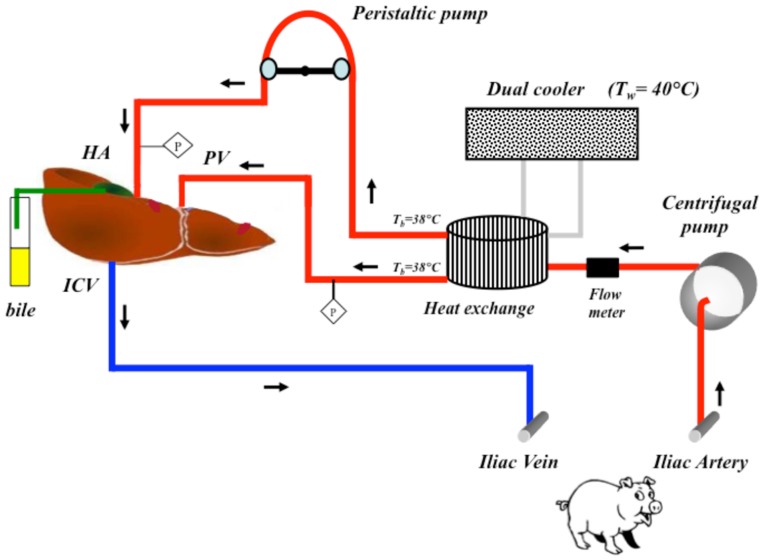
Schematic of the liver perfusion circuit. Representation of the *ex vivo* liver perfusion circuit as described in methods. HA: hepatic artery; PV: portal vein; ICV: inferior cava vein; Tb: blood temperature; Tw: water temperature; P: pressure transducer.

**Figure 2:**
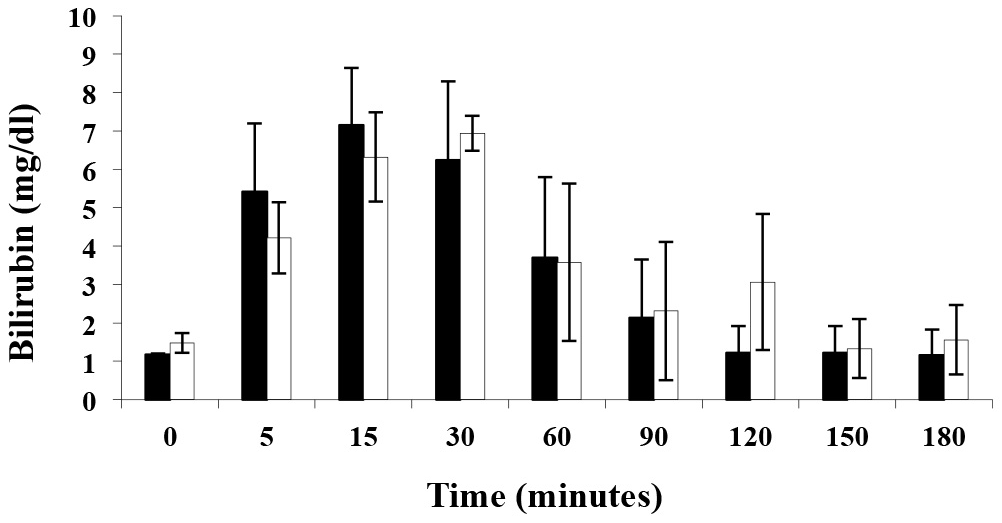
Kinetics of serum bilirubin levels in pigs in response to intravenous biliverdin administration. Biliverdin was administered as a single i.v. bolus of 50 µmol/kg. Results are mean ± SD of 3 pigs/treatment group. The black bars correspond to the donors and the open bars to the recipient.

Two treatment groups of pigs (n = 3) were used in this study. Pigs were acclimatized for 24 hours and given free access to food and water up to 12 hours before surgery. A control group, which received sham treatments and a biliverdin group where both donor and recipient pigs were administered a single bolus of biliverdin (50 µmol/Kg; Frontier Scientific B655-9 LY04-132) 2 hours prior to surgery. No further dosing was performed as this was based on effective dosing regiments performed in rodents. After the pre-treatment time with each molecule, recipient pigs were connected through an extracorporeal circuit to the isolated liver of the donor that was recovered and prepared as described below and shown schematically in ***Figure 1***. Serum bilirubin concentrations were monitored respectively for 4 and 3 hours post administration (***Figure 2***).

### Donor Pigs

Pigs were pre-medicated by intramuscular injection of Zoletil (5 mg/kg). Marginal veins in both ears were then cannulated for anaesthetic administration and solutions infusion. Anaesthesia was induced by Propofol (3–6 mg/kg, i.v.) and Ketamine (15 mg/kg i.v.). Butorphanol was administered by intramuscular injection (0.1–0.3 mg/kg) and by intravenous infusion (0.1–0.3 mg/kg) during the anesthesia induction and as needed for the duration of the experiment. At the end of the experiment euthanasia was induced by a slow infusion of Tanax (3 ml/10Kg). The liver recovery and warm dissection averaged 30–45 minutes. Briefly, following a median laparotomy, the common bile duct was cannulated with a 7 Fr sonde as distal as possible, near the duodenum and then distally ligated and transected. The porta was dissected with ligation and sectioning of two to three pancreatic branches including the splenic and superior mesenteric veins. The inferior cava under the liver was dissected from the parietal peritoneum just over the renal vein confluence and behind the liver. The animal was then heparinized (100 mg of heparin) and the porta was clamped and cannulated just over the splenic and superior mesenteric veins and the liver was flushed with cold 4°C Ringer solution. The hepatic graft was removed from the abdomen and packed in ice.

After sufficient cooling and flushing with approximately 2 liters of cold Ringers solution, the liver was flushed with 1 liter of cold Celsior® solution (Genzyme, Vienna, Austria). During perfusion the hepatic artery was also cannulated and the infrahepatic vena cava was closed. The suprahepatic vena cava was then cannulated with a 20 Fr cannula and all diaphragmatic veins (typically three) were closed with ligatures. Grafts weighed 505±75 grams. In addition, aminocaproic acid (65 mg/kg) was administered to the donor pig before surgery and to the isolated liver at 4 and 8 hours after reperfusion.

### Recipient Pigs

Two hours prior to surgery the recipients were administered BV as above. Animals were anesthetized two hours before being connected to the extracorporeal circuit. Following anesthesia, the right jugular vein and common carotid artery were cannulated for solution infusion, blood collection, and to measure central venous and mean arterial pressures (CVP and MAP) using disposable pressure transducer (Edwards Lifescience). After two hours a midline abdominal incision was made and systemic anticoagulation was started (100 mg of heparin, i.v.) and then administered every two hours to maintain the activated clotting time between 150 and 200 sec. The right external iliac artery and vein were cannulated for connection to the extracorporeal circuit. Approximately 19 hours (18.8±0.7 hrs) after the recovering and cold preservation of the donor liver, the isolated organ was connected via the extracorporeal circuit and the cannula was positioned both in the iliac artery and vein.

### Perfusion Circuit for the Isolated Perfused Liver

Prior to connection of the isolated liver to the perfusion circuit, the Celsior® solution was flushed from the portal circulation using 1 L of Ringer’s lactate solution. The perfusion circuit consisted of two pumps, one heat exchanger and PVS tubing (¼ and 3/16 inch external – internal diameter; refer to ***Figure 1***). The portal vein was perfused at a rate of 0.5 ml/min/g and a mean perfusion pressure was maintained between 12 mm Hg and 18 mm Hg. The hepatic artery was perfused using a peristaltic pump at a steady mean pressure between 60 and 90 mm Hg. The liver was positioned at a height of ∼ 70 cm above the recipient to allow venous return from the isolated liver suprahepatic inferior vena cava into the recipient iliac vein by gravity. Throughout the experiment, the temperature of the recipient pig and of the blood entering in the liver was monitored and maintained at 37.5±1°C by the heat exchanger. The ex vivo liver perfusion was performed up to 12 hours.

### Biochemical and Functional Assessments

Urea synthesis, ammonia clearance and lactate production were evaluated taking blood samples from both the inflow and outflow of the isolated liver graft and expressed as change in concentration between the two measurements. Aspartate aminotransferase (AST) determination in plasma samples was done using a LXJ725 Beckman Coulter analyzer.

### Histological and Immunohistochemical Evaluation of Liver Biopsies

Three to five liver biopsies were collected from each isolated liver and formalin-fixed or cryopreserved before ischemia, after cold ischemia and 12 hours after reperfusion. For each paraffin block 4 µm-thick serial sections were prepared and stained with hematoxylin-eosin to assess morphological features and architecture. The acute inflammatory response was evaluated by measuring the number of polymorphonuclear granulocytes in each of 14 high power fields (HPFs) and expressed as a percentage among the total number of cells present in each field. Four semiquantitative categories were generated as follows. 0: no polymorphonuclear granulocytes in the HPFs evaluated; 1: <10% of the total cells in the fields; 2∶10–30%; 3: >30%. Each serial section was dewaxed, rehydrated and pre-treated with 3% hydrogen peroxide for 5 minutes to inactivate endogenous peroxidases. Incubation with primary antibodies was then carried out, at room temperature, with the following antibodies: anti-Ki67 (clone MIB-1, DakoCytomation -Denmark dilution 1∶50); anti cleaved caspase-3 (clone 5A1 rabbit, Cell Signaling Technology-Danvers MA, U.S.A. dilution 1∶100). Primary antibodies were detected using horseradish peroxidase. Negative controls were performed on serial sections using primary antibodies with non-immune serum. Results of the immunohistochemical staining were blindly evaluated separately by two observers. In the case of discrepancy in evaluation of the immunostaining, the corresponding slides were re-evaluated jointly and resolved by consensus. A minimum of 10 high-power fields for each section was randomly selected for microscopic examination. The immunohistochemistry was quantified as a percentage of positive cells among the total cells evaluated. We used the TUNEL assay (ApopTag, Chemicon International-U.S.A.) to confirm apoptosis via DNA fragmentation examination. Nuclear counterstaining was performed with DAPI. The results of the staining were evaluated separately by two observers using a fluorescent microscope (Leica DM-RA2) with the appropriate excitation and emission filter. Ten fields for each sample (400× magnification) were acquired by a Leica DC350F camera. Apoptosis was quantified as the number of positive cells among the total cells present in all the selected fields (1000×900 pixels) containing about 500 cells.

### Statistical Analysis

All values are presented as means ± standard deviation (SD). Statistical analysis of the differences between experimental groups have been performed using non-parametric Kruskal-Wallis 1-way ANOVA by ranks test, using a 1.73 version of Analyse-it software add-in for Microsoft Excel (Windows version). A p value of ≤0.05 was considered statistically significant.

## Results

### Elevations in Serum Bilirubin Levels Following Administration of Biliverdin

Biliverdin is very rapidly converted to bilirubin by biliverdin reductase, with maximum levels of bilirubin in the range of 2.5–3 mg/dl achieved at 5 min to 15 min after biliverdin administration in rodent studies. The equivalent dose of biliverdin used here achieves similar bilirubin levels to those achieved in biliverdin-treated rodents. However, the time of maximum bilirubin levels after biliverdin administration was quite a bit later. Bilirubin in our studies was measured at the times indicated and peaked between 15–30 minutes after biliverdin administration (***Figure 2***).

### Effects of Biliverdin on IRI-induced Alterations in Liver Function

**Figure 3:**
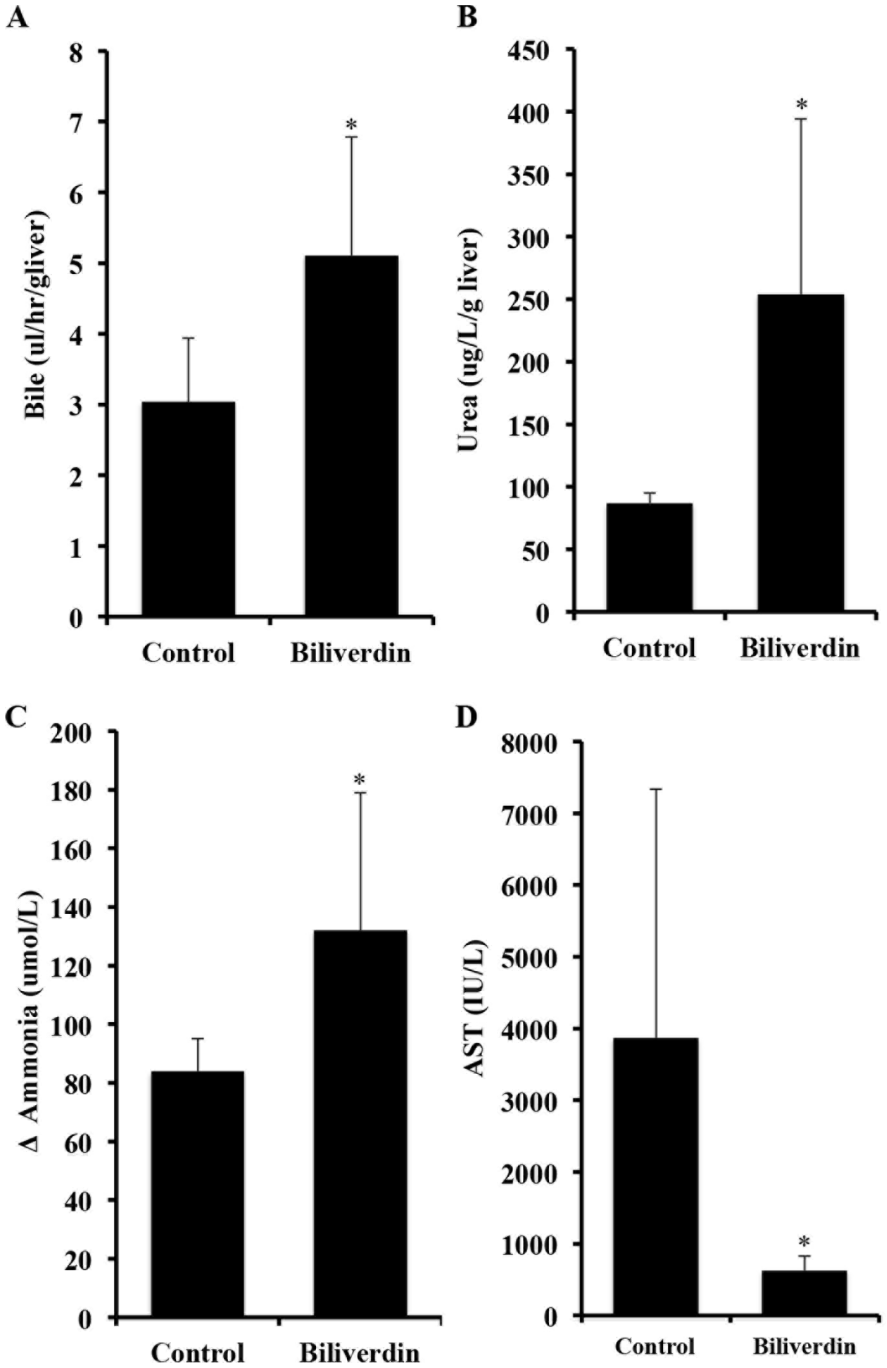
Effects of biliverdin on IRI-induced liver dysfunction. **A**. Biliverdin was administered separately to both donors and recipients before surgery. Bile production as a measure of liver function was collected throughout the experiment and expressed as µl/hr/g liver. Control pigs show very little bile production during 12 hours of reperfusion. Note that biliverdin significantly improved bile production and thus is indicative of better liver function. Results are expressed as mean ± SD from 3 pigs/group. The increase in bile production is statistically significant comparing biliverdin vs Ctrl *p = 0.03. **B**. Effects biliverdin on urea production. Urea synthesis is expressed as the difference (Δ) of the urea concentration as µg/L/g liver. Results are expressed as means ± SD of 3 pigs/group. *p = 0.022 **C**. Effects of biliverdin on ammonia clearance. Biliverdin was administered as described above. Ammonia was measured in the serum and the clearance is expressed as a difference (Δ) in ammonia in µmol/L between the inflow and outflow ports of the perfused liver. Results are expressed as mean ± SD of 3 pigs/group. The Δ ammonia clearance is statistically significant between the biliverdin treated vs Ctrl groups, *p = 0.027. **D**. Effects of biliverdin on serum AST levels**.** Venous blood samples were taken before and every 2 hours after graft reperfusion and expressed as calculation of the total amount of serum AST released throughout the 12 hr experiment. Livers from untreated controls showed a significant increase in AST levels indicating severe liver damage. Administration of biliverdin prevented the damage and release of AST into the serum vs Ctrl *p = 0.021. Results are mean ± SD of 3 pigs/group.

Function of the isolated liver was assessed by analyzing blood samples collected for bile production, urea and ammonia clearance and lactate accumulation both before entering the isolated perfused liver and at various times afterwards. Compared to livers of BV-treated animals, livers from controls showed a time-dependent decrease in liver function as indicated by lower bile production, urea and ammonia clearance (***Figures 3A-3C***) and lactate accumulation (*data not shown*). Liver damage was assessed by measuring the transaminase AST in the serum. Control livers showed a significant elevation in AST after 12 hrs of reperfusion, which was essentially completely abrogated in animals pretreated with biliverdin (***Figure 3D*** *p<0.05).

### Biliverdin Prevents IRI-induced Cell Death and Neutrophil Influx in the Liver

**Figure 4:**
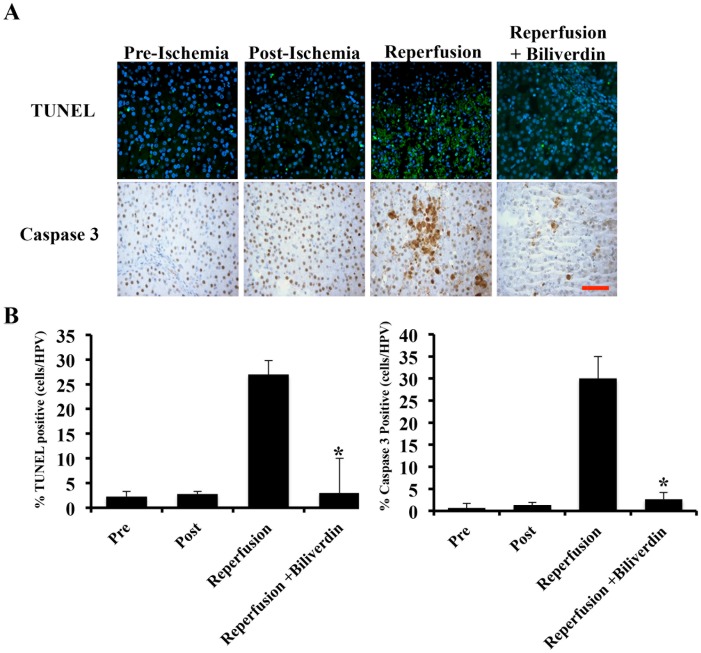
Effects of biliverdin on hepatocyte cell death. **A**. Representative immunostained liver sections for TUNEL and caspase 3 from liver sections harvested pre-ischemia, immediately post-ischemia and 12 hrs after reperfusion ± BV treatment. **B**. Quantitation of the number of positive cells in each stained sections as described in the methods. The degree of apoptosis and caspase 3 positivity was quantified by counting the number of positive cells among the total cells present in at least 10 selected fields with a minimum of 500 total positive cells counted. There is a statistically significant difference after 12 hrs of reperfusion *p = 0.01 versus ischemia alone and between biliverdin preconditioned animals after 12 hrs of reperfusion compared to controls (*p = 0.017). Results represent mean ± SD of 10 fields from 3 pigs/group where a total number of cells counted was at least 500. Magnification = 400×, Bar represents 50 µm.

To assess the protection afforded by biliverdin we studied IRI-induced apoptosis by TUNEL and caspase-3 activation. IRI increased both TUNEL and Caspase-3 activation after 12 hours of reperfusion (2.5±1.4%) in respect to before or just post ischemia. Biliverdin administration showed no TUNEL or Caspase-3 positivity (***Figure 4A–B***).

**Figure 5:**
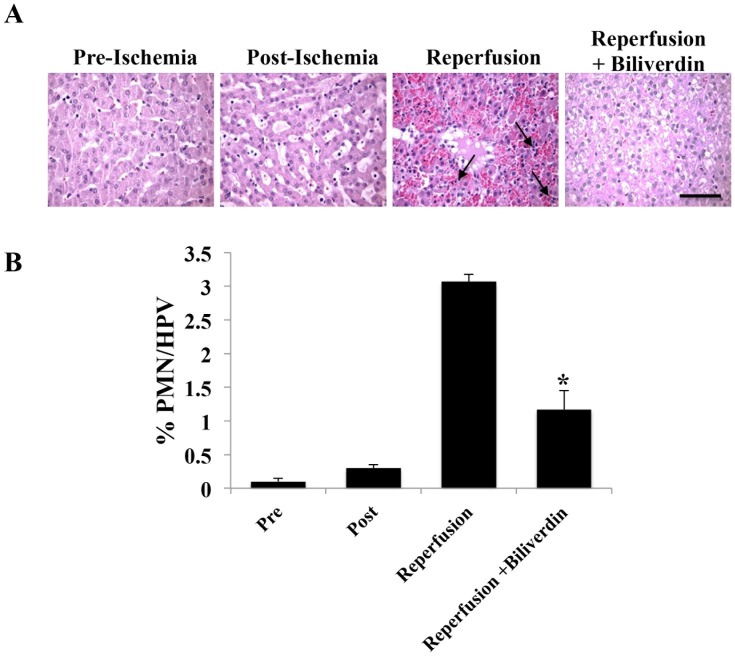
Effects of Biliverdin to Reduce Neutrophil Influx into the Liver. **A.** Representative H&E staining of liver sections harvested pre-ischemia, immediately post-ischemia and 12 hrs after reperfusion ± BV treatment. **B.** Neutrophils were counted based on morphology and expressed as a percentage of neutrophils among the total number of cells present in each field. Data are expressed as mean ± SD of n = 3 pigs/group where a total number of cells counted was at least 500. Magnification = 400×, Bar represents 50 µm. (*p = 0.013).

IRI also led to a rapid and significant increase in neutrophil infiltration into the liver that peaked 4–8 hour after reperfusion (4 h shown) in response to IRI and decreased to baseline after 12 hours of reperfusion (data not shown). Biliverdin significantly reduced >50% the neutrophil infiltration at both 4 hrs and 8 hrs after reperfusion vs controls (***Figure 5A–B***).

### Biliverdin Administration Increased Proliferation in the Liver after IRI

**Figure 6:**
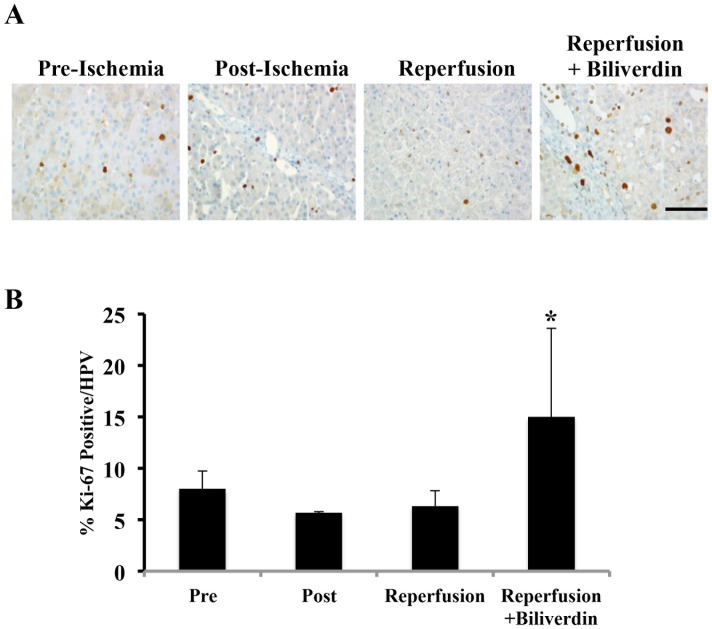
Biliverdin Treatment Increases Hepatocyte Proliferation After IRI. **A.** Representative immunostaining images for Ki-67 as a marker of cell proliferation in liver sections harvested pre-ischemia, immediately post-ischemia and 12 hrs after reperfusion ± BV treatment. Effects of biliverdin on Ki-67 expression as an indicator of cell (primarily hepatocyte) proliferation. **B.** Ki-67 positive cells were counted and expressed as a percentage of positive cells among the total number of cells present in each field. Data are expressed as mean ± SD of n = 3 pigs/group where a total number of cells counted was at least 500. Magnification = 400×, Bar represents 50 µm. (*p = 0.046).

Given that the liver is efficient at regenerating after insult, we also evaluated the effect of biliverdin on the cellular proliferation index (P.I.). Tissue sections were immunostained for Ki-67 (clone Mib-1) as an indicator of cell proliferation. Biliverdin-treated pigs showed augmented Ki-67 expression, suggesting that the liver was undergoing regeneration following the IRI insult (***Figure 6A–B***). These findings support the concept that BV is hepatoprotective following acute insult.

## Discussion

IRI is a common problem in medicine that is injurious in several situations including organ transplantation. Others and we have focused on transplantation studies in rodents to study IRI given the ready availability of the models and the importance of the problem in clinical transplantation. There is strong evidence correlating IRI with later problems of organ graft survival. It has thus been in the interest of the transplant physician to overcome this problem. This has been especially true as the need for organs has expanded and the use of marginal donor organs continues to expand. Primary non-function after transplantation is a major problem and particularly in the instances of marginal donor organs, which suffer from significant damage due to IRI.

It has long been accepted that “pre-conditioning” suppresses IRI [16]. Pre-conditioning involves exposure of the recipient to donor cells or other substances, in low numbers/amounts, a few days prior to transplantation of the organ [16]. Such manipulations have been shown to reduce IRI. While not well understood, there are a few reports that have shed light on the mechanisms by which pre-conditioning achieves its salutary effects [16]. However, to date pre-conditioning has not found acceptance in clinical practice. Interestingly, some of the changes seen with pre-conditioning and to which success is attributed, are also seen when HO-1, CO or biliverdin are used as therapeutics. These include, among others, increases in anti-inflammatory cytokines such as IL-10, anti-apoptotic proteins, such as inhibitor of apoptosis (IAP) and nuclear factor-kappa beta (NF-κB, 17) as well as heat shock proteins, such as HSP70 [18]–[21]. It may be that HO-1 induction or CO and biliverdin administration are effective because they mimic pre-conditioning, although there is no direct evidence for drawing any such parallels.

We demonstrate here in a unique model of liver IRI in pigs that biliverdin suppresses IRI of the liver. Swine are an accepted species on the basis of studies in which human testing might be undertaken. Data show clear salutary effects and that biliverdin in every case, proved significantly beneficial in the majority of the tests we did. Biliverdin proved to be potent cytoprotective agent that also reduced the infiltration of neutrophils and tissue damage significantly. We did not perform dose ranging studies, and thus the single does that was tested cannot be defined as optimized as it may well be that multiple doses and lower doses would be more effective and reduce any potential side effects of biliverdin. Biliverdin and bilirubin have been thought to act primarily via their anti-oxidant actions. We have recently found that biliverdin can also act in an anti-inflammatory manner by binding cell surface biliverdin reductase and interfering with TLR4 signaling as well as initiating signaling via PI3 kinase and Akt [22]–[23]. In these reports we delineate a novel localization and function for BVR on the cell surface, which is phosphorylated in response to BV or a stressor and rapidly, through an eNOS-dependent mechanism, translocates to the nucleus to regulate gene transcription [23].

In this study we treated the donor animal and the recipient with biliverdin that was rapidly converted to bilirubin in the serum. Whether the bilirubin that was generated was conjugated or unconjugated to albumin was not determined in these studies, but based on rodent studies where BV to BR results in elevations in conjugated bilirubin, we would expect a similar occurrence in pigs [24]. In rodents in a model of IRI in the heart, treatment of the donor and the recipient had beneficial results and even better effects when combined with inhaled carbon monoxide. CO has been extensively studied in transplant models [25]–[28]. Others have found that a biliverdin or bilirubin given just before transplantation but after preservation also had beneficial results [29]–[30]. Still others have induced HO-1 only in the donor and shown benefit from such treatment. In that case, however, one might argue that HO-1 is still expressed in the organ after transplantation and thus it is harder to ascertain where the effect is most important. From a clinical perspective, one could treat at all three stages. However, further experiments are indicated to dissect if donor treatment alone, for instance, will provide much of the effect seen when treatment is also given to the organ and then to the recipient.

Studies with biliverdin and bilirubin show enhanced survival of islets after allogeneic transplantation when treating only the donor leads to long-term (>100 days) survival and antigen-specific tolerance in a majority of the untreated recipients [31]. We have suggested that survival occurs because donor treatment leads to a very significantly diminished inflammatory response in the islets after transplantation, and thus less of an immune rejection response. A similar effect in organ transplantation might have profound consequences.

Biliverdin should be considered as potentially therapeutic in humans after appropriate safety and toxicology. The fact that biliverdin is essentially a biologic and thus considered a natural substance to the body would be expected to be relatively safe with a predictable pharmacokinetic and pharmacodynamic profile. Bilirubin, which is rapidly generated from biliverdin (***Figure 2***), can exhibit toxic effects at high concentrations in neonates, but appears to show no toxicity in adults at the concentrations we induce by the biliverdin doses recorded here. Importantly, individuals with Gilbert’s syndrome have bilirubin levels (<5 mg/dl) for their entire lives that are equal to or greater than those we find are therapeutic. Thus, elevating bilirubin to the levels found after biliverdin administration is unlikely to have undesirable side effects. Further, there is a strong association between high normal or supranormal (as in individuals with Gilbert’s syndrome) levels of bilirubin and less atherosclerosis-type disease as compared with individuals with low normal bilirubin levels [32]–[33]. These latter findings argue that raising bilirubin levels just a few folds may generally be helpful for health. It would thus appear that biliverdin exhibits a therapeutic window of efficacy versus toxicity that supports its therapeutic use in humans.

In summary, we present data here that demonstrate in a large animal model of prolonged liver ischemia reperfusion injury that a single pretreatment with biliverdin abrogates tissue inflammation and cell death and works toward normalizing hepatic function. We conclude with these data in a relevant preclinical model of IRI that either biliverdin is potential prophylactic agent to be tested in the clinical setting of organ transplantation and other indications that involve ischemia/reperfusion insults.
